# Dating violence and associated factors among male and female adolescents in Spain

**DOI:** 10.1371/journal.pone.0258994

**Published:** 2021-11-10

**Authors:** Carmen Vives-Cases, Vanesa Pérez-Martínez, MCarmen Davó-Blanes, Miriam Sánchez-SanSegundo, Diana Gil-González, Daniel G. Abiétar, Francesca Sánchez-Martínez, Lluís Forcadell-Díez, Glòria Pérez, Belén Sanz-Barbero

**Affiliations:** 1 Department of Community Nursing, Preventive Medicine and Public Health and History of Science, University of Alicante, Alicante, Spain; 2 CIBER de Epidemiología y Salud Pública, CIBERESP, Madrid, Spain; 3 Department of Health Psychology, University of Alicante, Alicante, Spain; 4 Barcelona Agency of Public Health, Barcelona, Spain; 5 Universitat Pompeu Fabra, Barcelona, Spain; 6 Institut d’Investigació Biomèdica (IIB Sant Pau), Barcelona, Spain; 7 National School of Public Health, Carlos III Institute of Health, Madrid, Spain; University of Westminster, UNITED KINGDOM

## Abstract

**Background:**

Dating Violence (DV) is a public health problem that is on the rise. In this paper, we aim to analyse different factors associated with DV victimization among female and male adolescents in Spain, considering socioeconomic circumstances, sexual orientation and the presence of different attitudes and experiences related to violence.

**Methods:**

Cross-sectional data from a convenience sample of 640 ever-partnered adolescents aged 13 to 17 at schools in the cities of Alicante (n = 359, 50.1% girls) and Terrassa (n = 281, 51.9%) in the context of an educational intervention to promote healthy relationships. We calculated the prevalence of different forms of DV (physical, sexual and control and fear) and carried out multivariate regression models by sex.

**Results:**

5.5% of girls and 8.7% of boys declared having suffered lifetime physical and/or sexual violence, while 22% of girls and 20.5% of boys reported control and/or fear victimization. The likelihood of DV was higher among migrants and those with foreign-born parents (aPR girls = 2.1 CI95%: 1.1–3.9; aPR boys = 1.9: CI95%: 1.0–3.6); prior experiences of abuse (aPR girls = 1.6; CI95%: 1.0–2.6; aPR boys = 1.7; CI95%: 1.1–2.6); and those who showed higher levels of machismo (aPR girls = 1.0; CI95%: 1.0–1.1; aPR boys = 1.0; CI95%: 1.0–1.1). In girls, DV increased among those who reported lesbian/bisexual orientation and poor relationship with teachers.

**Conclusions:**

DV is socially patterned and increases among LGB adolescents (especially in the case of girls), migrants, and those with foreign-born parents, and adolescents who reported prior experiences of violence in childhood. Future DV prevention programs should consider social inequalities in the likelihood of DV and by reinforcing adolescents’ abilities to recognize social support sources and reject machismo and violence.

## Introduction

Teen Dating Violence (DV) refers to physical, sexual, psychological, and stalking behaviours that occur in the context of a close relationship between teenagers. DV is considered to be a type of intimate partner violence [1]. New concerns are emerging due to its damaging short and long-term effects on teenagers’ health and wellbeing [2]. In the international context, it has been estimated that the prevalence of physical violence among adolescents aged 13 to 18 is 20% and that the prevalence of sexual violence is 9% [3]. According to data from the last Macro Survey on Gender Violence in Spain [4], 46.6% of women aged 16–24 that have had a partner declared having experienced some type of violence on behalf of that person. The prevalence in women over age 25 was 32.4%. DV victimization is also common among male adolescents, although its consequences seem to be worse for girls [2].

There is also evidence that shows that social and economic factors are associated with a higher risk for DV, such as being of older age, experiencing lower socioeconomic conditions and belonging to a minority ethnic group [5, 6]. An increased risk of DV victimization has been also reported by female and male adolescents who have been exposed to other forms of violence (childhood exposure or witnessing different forms of violence, bullying), poor quality friendships and family relationships, and the presence of harmful attitudes such as sexism, machismo, or violence acceptability [7–10]. Conversely, a higher sense of attachment to school and teachers seems to be associated with a lower likelihood of both DV victimization and perpetration [11].

The influence of sexual orientation and identity on DV is an emerging field. Research has shown that LGB youth (lesbian, gays, bisexuals) may have a higher risk of physical and/or sexual DV victimization than heterosexual adolescents [12]. According to the U.S. National School Climate Survey, nearly 85% of LGB/Transgender students have experienced verbal harassment, and 27% have been physically harassed at school [13]. The most common form of violence includes the use of homophobic language and/or spreading sexual orientation rumours or mocking students who are perceived as non-normative compared to a monogamic heterosexual norm [14, 15]. Research has shown that the likelihood of DV may be higher among male and female adolescents who are not sure of their sexual identity [16].

Although there is some prior research, the evidence is still weak concerning DV among both male and female adolescents in Europe [17–19]. There is a need for studies that integrate the wide variety of protective factors and potential precursors to DV, which may contribute to public health strategies to prevent DV and promote healthy and equitable relationships.

During the 2019–2020 period, we conducted an educational interventional project titled “Promotion of Gender Violence Protective Assets Among Adolescents and Pre-adolescents” in secondary education schools. The project was funded by the Spanish Ministry of Economy, Industry and Competitiveness and the Carlos III Institute (Ref. PI18/00590 and PI18/00544) in 2019 and 2021. This project was based on a prior European project: Lights, Camera and Action against Dating Violence -Lights4Violence, an educational intervention carried out in six European countries to promote positive relationships among adolescents [20]. In the latter project, the educational intervention program aimed to promote personal and external dating relationship assets among a sample of adolescents ages 13–18 from two Spanish cities, Alicante and Terrassa (Barcelona), both of which are located on the Mediterranean coast of Spain.

In this paper, we used the baseline data to analyse the prevalence and different factors associated with DV victimization among female and male adolescents schooled in two cities of Spain, considering their socioeconomic circumstances, sexual orientation and the presence of different violence-related attitudes and experiences in 2019–2020.

## Materials and methods

This study had a cross-sectional design. A total of eight high schools participated (6 public, 2 subsidized), with 35 classes from the 2nd course and 34 classes from the 3rd course (years 9 and 10 in secondary school, respectively). The selection of the schools was carried out by contacting different secondary schools that met the characteristics of the study (non-random sample). We estimated that this number of schools would allow us to reach the estimated sample size for our intervention. In order to calculate the sample size, we used the GRANMO software. Calculating a rate of losses to follow-up of 20% and with an alpha risk of 0.05 and a beta risk <0.02 in a bilateral contrast, we estimated that 279 subjects (CG) would be needed for the intervention group and 279 subjects in the control group to detect a statistically significant difference between two proportions (expected to be 0.1 for the intervention group and 0.2 for the control group). Lost to follow-up ratio was estimated at 30%, as is usually expected in studies with these characteristics.

From among the total of the students (n = 1,846) at the selected high schools, 1,561 (84.6%) were offered the opportunity to participate in the survey because they were present at school. Of those invited, 1,538 students and their teachers accepted (98.5% of those contacted). The final study population of the baseline survey was 1,422 students (91% of those invited). In this study we selected 640 high school students ages 13 to 17 (51.3% girls), who reported having had a partner. The sample was selected from the four schools in Alicante (n = 359, 50.1% girls) and the four in Terrassa, Barcelona (n = 281, 51.9% girls).

Data was collected through an online questionnaire distributed to the different schools in Alicante and Terrassa between October 2019 and February 2020. Surveys were personally and confidentially self-administered. The students answered the questionnaire on separate computers with total privacy. Members of our research team were present in the classroom throughout the survey (which lasted approximately one hour). This facilitated access to the survey, helped resolve possible language barriers and ensured that each participant answered their questionnaire confidentially. The teachers were asked to remain outside the classroom, although this was not always possible. Methodological details of the survey are based on previously published study [20].

The students from the eight centres included in the project agreed to participate, through prior informed consent for them and their legal guardians. The project was approved by two ethical committees, CEIm-Parc de Salut Mar (2019-8914-I) and CEIC Alicante University (UA-2018-02-28).

### Measures

Victimization of DV was used as a main outcome of the study [21, 22]. Students were asked about their possible exposure to situations of physical and sexual abuse and control and fear. The questions about physical and sexual violence were: “Has anyone that you have ever been on a date with physically hurt you in any way? (For example, slapped you, kicked you, pushed, grabbed, or shoved you)”; “Has a person that you have been on a date with ever attempted to force you to take part in any form of sexual activity when you did not want to?”; The questions about fear and control were: “Have you ever perceived your partner’s control of your daily activities?” and “Have you ever been threatened or felt fear because of your partner’s behaviour?” Using the gathered responses, a dichotomous variable was created with the categories of “physical and sexual violence” and “control and fear”.

Different sociodemographic variables were also collected related to students’ sex, age, country of birth and nationality and their parent’s nationality, employment, and education level. Parents’ employment was classified as “paid work” and “unpaid work”. The “unpaid work” option integrated the following responses: homemaker (exclusively), unemployed, retired, and unable to work because of a disability, student, died, don’t know. The educational level was classified as “primary studies or lower” and “secondary studies or higher”. This last option was made up of secondary school, vocational training, or university.

Sexual orientation was reported according to Kinsey’s scale [23], as “with which of the following phrases do you feel most identified?” The answers possible were”I feel only attracted to people of my same sex”, “normally I feel attracted to people of my same sex, but sometimes I feel attracted to people of a different sex”, “I feel attracted to people of my same sex and of a different sex”, “indifferent”, “normally I feel attracted to people of a different sex, but sometimes I feel attracted to people of my same sex”, “I feel only attracted to people of a different sex”, “I’m not sure/I don’t feel attracted to anyone”. We collapsed all answers into two categories: heterosexual (“I feel only attracted to people of a different sex”) and “others” (including those LGB, those that they don’t know and those that are not attracted to anyone).

We also asked about prior experiences of abuse and/or violence in childhood before age 15 using the following three questions [24]: “Before you were 15 years old, did any adult -that is, someone 18 years or older- physically hurt you in any way? (For example, slapped you, kicked you, pushed, grabbed, or shoved you)”; “Before you were 15 years old, did someone 18 years or older force you to participate in any form of sexual activity when you did not want to?”; “Before you were 15 years old, did you witness someone in your family environment (your father or your mother’s partner) physically beat or mistreat your mother?”

Social support was assessed through three questions related to support from parents, classmates and at high school, with five Likert response options for parents and classmates (1 “very good” to 5 “very bad”) and four Likert response options for high school (1 “I like so much” to 4 “I don’t like”). Questions asked were “What are your relationships with your family like? Referring to the people you live with”, “How are the relationships with your classmates usually?” and “Do you like your high school?” [25].

Machismo and violence acceptability were measured using the Maudsley Violence Questionnaire [26]. This scale is made up of 56 items with a dichotomous scale (true or false). This scale evaluates violent thinking through two subscales, one of which is machismo (42 items; range 0–42), which includes aspects related to the importance of being violent and strong for manliness, and the association of weakness or embarrassment with non-violence or backing down. Acceptance of violence (14 items; range 0–14) is the second subscale and evaluates aspects about the enjoyment of violence and injunctions against or rejection of violence as an acceptable behaviour [26].

Sexism was measured using the Ambivalent Sexism Inventory (ASI) [27], a scale made up of 22 items that measures the level of agreement in six categories using a Likert-type scale ranging from 0 (total disagreement) to 5 (total agreement). The ASI consists of two subscales with 11 items each: Benevolent Sexism and Hostile Sexism. Higher scores indicate more sexist behaviour (Glick & Fiske 1996).

Bullying/cyberbullying was measured through the adapted version of the Lodz Electronic Aggression Questionnaire (LEAQ) [28]. This tool measures bullying and cyberbullying, understood as serious forms violence among adolescents that is regular, intentional and involves an imbalance of power and includes involvement of a perpetrator and a victim, also in the context of current or former romantic partners. The four questions referred to the last three months, and the scale included Likert answers (never—3 times or more).

### Data analysis

First, we carried out a descriptive analysis of the sample for each of the variables used in the study to observe the prevalence of global dating violence and for each of the types of violence (physical and sexual, control and fear). Second, a chi-square test (categoric variables) and Student t test (quantitative variables) was used to assess whether there were differences in the prevalence of dating violence for each of the study variables. Lastly, prevalence ratios were calculated (PR) using Poisson regressions with robust variance using dating violence as the outcome variable. The selection of variables was carried out through a forward stepwise procedure to explore which variables could add more significance to the model. The selected variables were also included in previous studies concerning the factors associated with dating violence [10, 29]. The significance level considered in all the analyses was >.05. All the analyses were stratified by sex and used the Stata 14.0 software for the data analysis.

## Results

**Fig 1:**
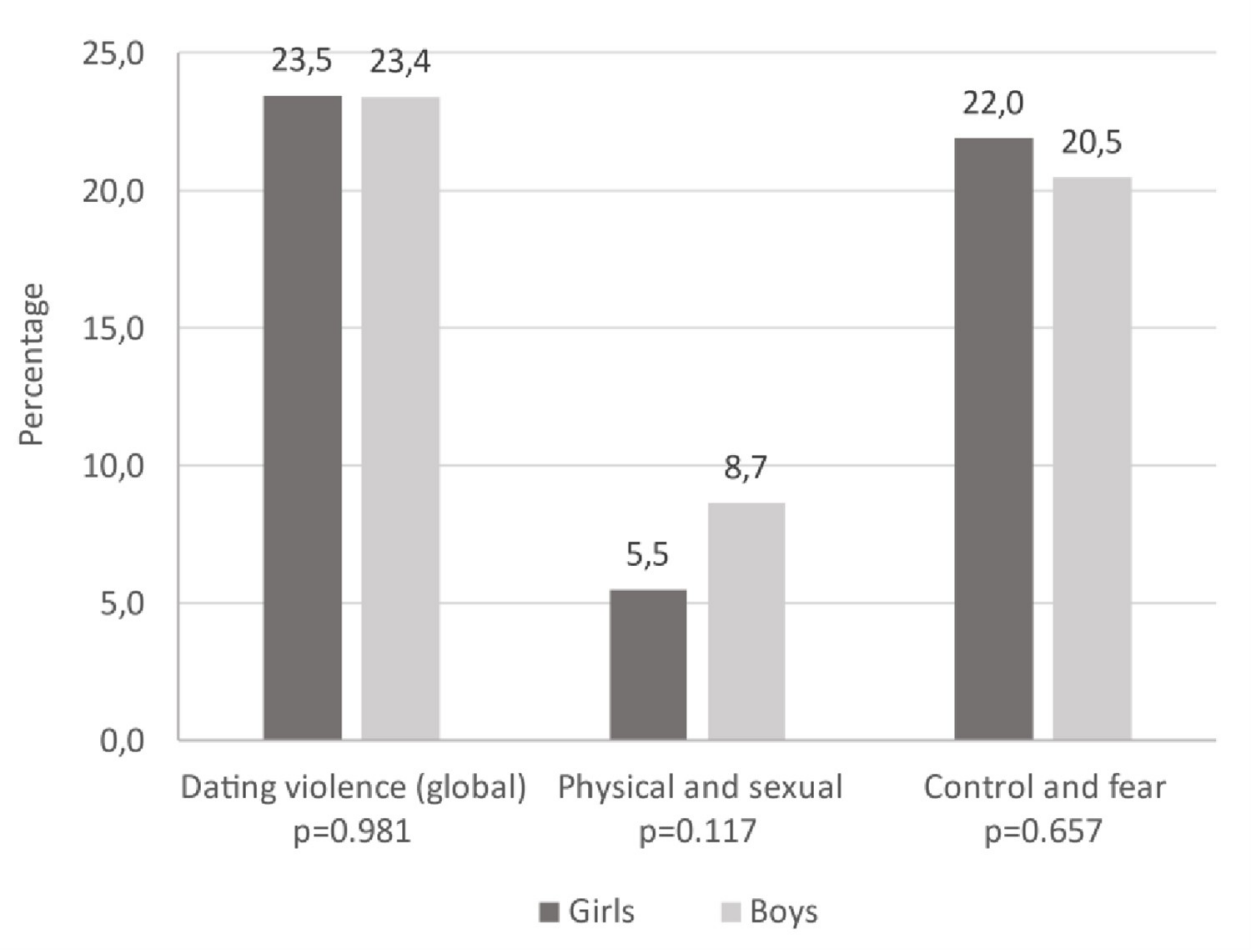
Prevalence of violence by sex.

The prevalence of DV was 23.5% in girls and 23.4% in boys. Around 5.5% of girls and 8.7% of boys reported having experienced physical and/or sexual violence at some time in their lives. About 22% of girls and 20.5% of boys reported having experienced violence related to control/fear (Fig 1).

Heterosexual girls presented lower prevalence of DV (19.3%) than girls with other types of sexual orientations (36.7%) p = 0.004. In boys, the prevalence of DV for those born in a foreign country was 43.8%, 28.1% in boys born in Spain with foreign-born parents and 19.0% for those born in Spain (p = 0.005) (Table 1).

**Table 1:** Prevalence of teen dating violence victimization by sex according to sociodemographic characteristics and variables related to exposure to violence.

Variable	Girls			Boys		
	Total	Prevalence dating violence	p-value*	Total	Prevalence dating violence	p-value*
	n (%)	n (%)		n (%)	n (%)	
Total	328 (100.0)	77 (23.5)		312 (100.0)	73 (23.4)	
Age			0.120			0.113
15–18 years	58 (17.7)	18 (31.0)		49 (15.7)	14 (28.6)	
14 years	155 (47.3)	29 (18.7)		150 (48.1)	40 (26.7)	
13 years	115 (35.1)	30 (26.1)		113 (36.2)	19 (16.8)	
Place of birth			0.110			0.005
Foreign	32 (9.8)	12 (37.5)		32 (10.3)	14 (43.8)	
Spain	217 (66.2)	50 (23.0)		216 (69.2)	41 (19.0)	
Spain, foreign parents	79 (24.1)	15 (19.0)		64 (20.5)	18 (28.1)	
Mother’s work status			0.885			0.358
Not working	83 (25.3)	19 (22.9)		65 (20.8)	18 (27.7)	
Working	245 (74.7)	58 (23.7)		247 (79.2)	55 (22.3)	
Father’s work status			0.746			0.390
Not working	33 (10.1)	7 (21.2)		39 (12.5)	7 (17.9)	
Working	295 (89.9)	70 (23.7)		273 (87.5)	66 (24.2)	
Mother’s education			0.254			0.685
Primary or lower	98 (29.9)	19 (19.4)		84 (26.9)	21 (25.0)	
Secondary or higher	230 (70.1)	58 (25.2)		228 (73.1)	52 (22.8)	
Father’s education			0.312			0.163
Primary or lower	108 (32.9)	29 (26.9)		103 (33.0)	29 (28.2)	
Secondary or higher	220 (67.1)	48 (21.8)		209 (67.0)	44 (21.1)	
Desire orientation			0.004			0.765
Heterosexual	249 (75.9)	48 (19.3)		247 (79.2)	53 (21.5)	
Non-heterosexual and non- attraction	79 (24.1)	29 (36.7)		65 (20.8)	20 (30.8)	
Violence in childhood			0.001			0.001
No	235 (71.6)	44 (18.7)		232 (74.4)	43 (18.5)	
Yes	93 (28.4)	33 (35.5)		80 (25.6)	30 (37.5)	
Witness abuse in childhood			0.003			0.003
No	303 (92.4)	65 (21.5)		281 (90.1)	59 (21.0)	
Yes	25 (7.6)	12 (48.0)		31 (9.9)	14 (45.2)	
Cyberbullying victimization			0.021			0.011
No	292 (89.0)	63 (21.6)		261 (83.7)	54 (20.7)	
Yes	36 (11.0)	14 (38.9)		51 (16.3)	19 (37.3)	
Cyberbullying perpetration			0.027			0.070
No	316 (96.3)	71 (22.5)		292 (93.6)	65 (22.3)	
Yes	12 (3.7)	6 (50.0)		20 (6.4)	8 (40.0)	
Bullying victimization			0.003			0.016
No	278 (84.8)	57 (20.5)		263 (84.3)	55 (20.9)	
Yes	50 (15.2)	20 (40.0)		49 (15.7)	18 (36.7)	
Bullying perpetration			0.001			0.205
No	307 (93.6)	66 (21.5)		292 (93.6)	66 (22.6)	
Yes	21 (6.4)	11 (52.4)		20 (6.4)	7 (35.0)	

*Chi-square test.

We also observed that earlier experiences of abuse and/or violence in childhood prior to age 15 and having been a victim of bullying and cyberbullying were statistically significantly related to greater DV prevalence, in both girls and boys. In the case of girls, the prevalence of DV increased when they were aggressors in bullying or cyberbullying situations. Also, in both girls and in boys, there was greater average machismo and acceptance of violence when they had experienced DV. Furthermore, there were significant differences among girls in terms of family relationships (p = 0.021) and in terms of school satisfaction (p = 0.041) (Table 2).

**Table 2:** Teen dating violence victimization by sex according to sexism, machismo, empathy, assertiveness and capacity for conflict resolution.

Variable	Girls				Boys			
	Total	Yes dating violence	No dating violence	p-value*	Total	Yes dating violence	No dating violence	p-value*
	Mean (SD)	Mean (SD)	Mean (SD)		Mean (SD)	Mean (SD)	Mean (SD)	
Good relationship with family	1.8 (0.9)	1.9 (1.0)	1.7 (0.8)	0.021	1.7 (0.8)	1.8 (0.8)	1.7 (0.8)	0.122
Satisfaction with the school	1.8 (0.8)	2.0 (1.0)	1.8 (0.8)	0.041	2.0 (0.8)	2.1 (0.8)	1.9 (0.8)	0.206
Good relationship with teachers	2.1 (0.8)	2.2 (0.9)	2.1 (0.7)	0.181	2.2 (0.8)	2.4 (1.0)	2.2 (0.8)	0.089
Good relationship with classmates	1.6 (0.7)	1.7 (0.7)	1.6 (0.7)	0.592	1.7 (0.7)	1.8 (0.8)	1.6 (0.7)	0.105
Violent Thinking total	14.4 (9.3)	16.5 (9.2)	13.8 (9.2)	0.026	15.0 (9.2)	17.2 (10.6)	14.3 (8.6)	0.019
Machismo	8.9 (6.8)	10.7 (7.0)	8.4 (6.6)	0.009	9.3 (7.0)	11.5 (8.1)	8.7 (6.5)	0.003
Violence acceptability	5.5 (3.4)	5.8 (3.1)	5.4 (3.5)	0.387	5.7 (3.2)	5.8 (3.4)	5.7 (3.2)	0.817
Global sexism	47.9 (10.8)	48.2 (10.9)	47.7 (10.8)	0.714	48.5 (11.9)	50.1 (12.6)	48.0 (11.7)	0.181
Hostile sexism	22.9 (6.4)	23.4 (6.4)	22.8 (6.4)	0.411	23.4 (6.6)	24.4 (7.2)	23.1 (6.5)	0.135
Benevolent sexism	24.9 (6.6)	24.8 (6.7)	25.0 (6.6)	0.847	25.1 (7.0)	25.7 (7.0)	24.9 (7.0)	0.393

*Student t-test; SD: Standard deviation.

Girls who were born in a foreign country had 2.1 times greater probability of experiencing DV than those who were born in Spain and had foreign-born parents (confidence interval (CI) 95%: 1.1–3.9). Having an LGB sexual orientation was also associated with a greater risk of DV in girls (aPR: 1.9; CI95%: 1.3–2.9). Girls with prior experiences of abuse and/or violence in childhood presented a 60% greater chance of experiencing DV (aRP: 1.7; CI95%: 1.1–2.8). Greater machismo was also associated with greater probability of DV (aPR: 1.0; CI95%: 1.0–1.1), while greater acceptance of violence was associated with a lower probability of DV (aPR: 0.9; IC95%: 0.9–1.0). Those girls with a poor relationship with teachers also showed greater probability of experiencing DV (aPR: 1.6; CI95%: 1.1–2.3) (Table 3).

**Table 3:** Main associated factors of teen dating violence victimization among girls and boys.

	Girls				Boys			
	aPR*	CI 95%		p-value	aPR*	CI 95%		p-value
Alicante (Ref.)								
Barcelona	1.32	0.81	2.10	0.278	1.21	0.72	1.91	0.539
Age groups (Ref.: <13 years)								
15–17 years	1.14	0.72	1.92	0.629	1.52	0.81	2.92	0.204
14 years	0.85	0.50	1.23	0.272	1.54	0.93	2.63	0.096
Birthplace (Ref.: born in Spain, foreign parents)								
Foreign-born	2.11	1.12	3.90	0.018	1.95	1.04	3.63	0.038
Born in Spain	1.63	0.95	2.43	0.074	0.93	0.62	1.54	0.712
Mother’s education (Ref.: no higher education)								
Superior	1.58	1.00	2.54	0.076	1.12	0.73	1.73	0.771
Heterosexual (Ref.)								
Others	1.92	1.32	2.93	0.001	1.32	0.83	2.10	0.319
Physical and sexual abuse in childhood (Ref.: No)								
Yes	1.27	0.85	1.91	0.297	1.70	1.13	2.61	0.012
Witnessed abuse (Ref.: No)								
Yes	1.71	1.10	2.82	0.025	1.52	0.90	2.43	0.117
Machismo	1.05	1.02	1.11	0.002	1.04	1.01	1.17	0.017
Violence acceptability	0.93	0.91	1.00	0.025	1.02	0.91	1.10	0.401
Satisfaction with teachers (Ref.: good relationship)								
Bad relationship	1.62	1.10	2.39	0.018	1.20	0.80	1.80	0.388
Family relationship (Ref.: Good relationship)								
Bad relationship	1.46	0.92	2.29	0.159	0.84	0.51	1.52	0.547

Multivariate Poisson Regression Model with Robust Variance.

* Adjusted prevalence ratio.

Regarding the boys, those born in a foreign country presented a 90% greater likelihood of experiencing DV than those born in Spain with foreign-born parents (RP: 1.9: CI95%: 1.0–3.6). Those who experienced physical and/or sexual abuse showed a 70% greater likelihood of having experienced DV (aRP: 1.7; CI95%: 1.1–2.6). Greater machismo was associated with a greater likelihood of experiencing DV in boys (aPR: 1.0; CI95%: 1.0–1.1) (Table 3).

## Discussion

Nearly one fourth of students aged 13–18 reported having been exposed to physical-sexual violence and/or control and fear. Among them, the registered prevalence among lesbian/bisexual girls and those who have not defined their sexual orientation yet are noteworthy; as is that of boys who were born outside Spain and those born in Spain with foreign-born parents; and female and male adolescents with poor relationships with friends, family and/or teachers. Being foreign-born and scoring a higher level of machismo were common factors associated with an increased likelihood of DV among girls and boys. In addition, boys who suffered physical and/or sexual abuse in childhood were 70% more likely to have suffered DV, and girls who witnessed these abuses in childhood were 60% more likely. In the case of girls, other DV associated factors included being lesbian, bisexual or unsure about one’s sexual orientation and having a poor relationship with teachers.

In accordance with previous research [29, 30], DV victimization is present in both girls and boys. In the case of girls, data published by the European Union Agency for Fundamental Rights Violence Against Women Survey of 2012 shows similar prevalences to what we obtained in our study, despite the different methodologies used in both surveys [19]. A meta-analysis carried out by Wincentak [3] shows that, in the case of sexual DV victimization, the prevalence is significantly greater in girls (14%) than in boys (8%). In our study, however, the observed prevalence of DV among girls and boys was similar. We need to look at this from a feminist perspective, because in heterosexual relationships, the mechanisms and severity by which violence operates differ between the sexes. Thus, men’s violence against women escalates faster and is more socially legitimized than the reverse. In this sense, violence is directional. On the other hand, we must discuss how men and women perceive violence. Based on the literature, heterosexual women have normalized much of everyday violence, to that they do not perceive it as such. Men, on the other hand, clearly identify when women use control or aggression, and this often occurs in response or reaction to previous violence by men [31].

In relation to the observed DV associated factors in this study, worth noting is the greater probability of foreign-born girls and boys to experience dating violence, compared to the young population born in Spain. On the one hand, in interpreting this result, it is important to consider that the immigrant population may have different conceptualizations of love, and may be more tolerant of certain behaviours considered violent compared to the native-born population [32, 33]. On the other hand, the immigrant population from low-income countries that migrates for economic reasons may be exposed to greater social and economic inequality than the native-born population [34, 35]. These inequalities are expressed in the context of systematic discrimination, violence and poverty that condition social opportunities and intergenerational life circumstances of the immigrant population. The social and health situation of the immigrant population requires an intersectional approach, given that inequalities operate simultaneously and are complex in their economic, social and gender dimensions, among others [36].

In this study, we confirmed the important influence of previous experiences of violence (suffered or witnessed) on the likelihood of current DV victimization among both girls and boys [18, 29]. Research suggests that a violent family culture may provide youth a biased model of interpersonal interaction, which normalizes aggressive behaviours and may make boys and girls learn that physical and verbal coercion are adequate and acceptable strategies for changing someone else’s behaviour and solving conflicts in their dating relationships [37]. This shows how important it is to detect childhood abuse situations and implement interventions early to prevent, as far as possible, the development of violent attitudes during adolescence and adulthood [38].

The results of this study also found that DV victimization among both girls and boys was associated with higher levels of machismo. Machismo has been associated with an increase in the level of conflicts in a relationship, making adolescents more vulnerable to becoming victims of violence and aggression and more emotionally dependent to the relationship [9]. The results of our study also showed that the likelihood of DV victimization in girls decreases with higher levels of violence acceptation. Adolescents with a higher tolerance of violence may display lower awareness of their rights, which may in turn lead them to justify their actions [39]. This finding highlights the importance of interventions focused on training social and emotional skills in young people at high risk of machismo and acceptation of use of violence given these variables may operate as precursor of violence and victimization.

In agreement with prior research [2, 4], the prevalence of DV in both girls and boys was higher among LGB adolescents and those who are not sure of their sexual orientation. In addition, an association was also confirmed between DV and being lesbian, bisexual or unsure about one’s sexual orientation in the case of girls. The literature suggests that internalized homophobia could explain this association between non-heterosexual orientation and IPV victimization in girls [40]. The discrimination (based on the feelings/beliefs of others) related to sexual orientation is related to individuals’ own feelings regarding their orientation (i.e., stigma conscientiousness). Likewise, the psychosocial stress that LGB groups may experience in coping with repeated situations of discrimination and marginalization could also be a risk factor for IPV victimization [40]. Interventions that address violence during dating relationships should include information on heterosexual and non-heterosexual relationships and promote school support groups and LGB-heterosexual partnerships [41].

Those adolescents that reported higher prevalence in DV victimization were those who reported poor relationships with their close circles–family and school environment for girls and school environment for boys. In addition, the likelihood of DV victimization in girls was higher for those who had a poor relationship with the school’s teaching staff. This finding is consistent with previous studies that have shown that the likelihood of suffering physical and/or sexual DV decreased when school social support increased [10]. Other studies have shown a moderating effect of social support in terms of physical and psychological DV victimization and relationship satisfaction in girls [42]. These results evidence the importance of social support resources to which teens can turn to seek help [43].

There are some limitations that should be taken into account when interpreting these results. First, the sample used was not representative of both cities or of the country of Spain, and our sampling was non-probabilistic, as it was designed for a pilot school program (quasi-experimental study). Second, we were unable to collect information about students who refused to take part in the survey. Third, the cross-sectional design of our study did not allow us to identify a cause-and-effect relationship between the observed associated variables. Despite, these limitations, this study provides evidence of the risk factors associated with dating violence among girls and boys in Spain. We used a large sample with statistical power to detect significant differences, which may solidify our results. Also, the current study has important implications that may contribute to public health strategies to prevent DV and promote healthy relationships. Thus, implementing prosocial interventions focused on developing emotional and interpersonal skills among adolescent students may be important strategies to prevent risky behaviours, including violence and victimization. Likewise, it’s necessary to draw up strategies with a gender perspective, to address the different mechanisms through which boys and girls participate in heterosexual relationships with DV. These strategies should be a priority among vulnerable adolescents, including LGB and foreign-born adolescents, since these subgroups with high levels of DV may require additional support from community services.

## Conclusions

The magnitude of different forms of DV among girls and boys registered in this study shows the need for improvement in violence prevention programs and the promotion of healthy and equitable relationships. According to our results, DV is socially patterned and is worse among the foreign-born, adolescents who reported prior experiences of violence in childhood and LGB groups (especially in the case of girls) who must cope with experiences of social discrimination and harassment. Future intervention programs, such as those promoted by the Spanish State Agreement against Gender Violence in 2017 [44, 45], should be improved by considering these social inequalities in the likelihood of DV and by reinforcing adolescents’ abilities to develop skills and recognize different social support sources in their own close circles to cope with machismo.

## Supporting information

S1 File (PDF)

S2 File (PDF)
