# Supplementary material for: Dating violence and associated factors among male and female adolescents in Spain
Source: PLoS One. 2021 Nov 10;16(11):e0258994. doi: 10.1371/journal.pone.0258994 (PMC8580219; doi:10.1371/journal.pone.0258994)
Supplement: S1 File — (PDF) [file pone.0258994.s001.pdf]

ordenamos de menor a mayor AIC

Variable	N	Chi2	p-value	Log likelihood	AIC	BIC	
Nationality	325	2,07	0,151	-199,687	403,37	410,94	Elimino esta variable n los siguientes pasos por tener muchos perdidos
Child_fis_sex2cat	640	23,07	0	-359,837	723,67	732,6	
witness_abuse2	640	22,67	0	-362,101	728,2	737,13	
MVQ_MACHISMO	640	17,69	0	-362,211	728,42	737,34	
Bullying_victima	640	16,34	0	-362,777	729,55	738,48	
Ciberbullying_victima	640	13,32	0	-363,713	731,43	740,35	
SP_TOTAL	640	10,67	0,001	-363,713	731,43	740,35	
MVQ_TOTAL	640	11,15	0,001	-363,869	731,74	740,66	
AUT_TOTAL	640	10,15	0,001	-364,028	732,06	740,98	
Bullying_agresor	640	12,8	0	-364,462	732,92	741,85	
Satisfaccion_instituto_2cat	640	9,52	0,002	-364,563	733,13	742,05	
tipo_nac	640	14,02	0,001	-363,758	733,52	746,9	
Satisfaccion_profesorado_2cat	640	7,98	0,005	-364,77	733,54	742,46	
Relacion_familiar	640	8,1	0,004	-364,869	733,74	742,66	
Ciberbullying_agresor	640	9,79	0,002	-365,236	734,47	743,4	
Relacion_familiar_2cat	640	7,27	0,007	-365,278	734,56	743,48	
Satisfaccion_instituto	640	5,7	0,017	-365,601	735,2	744,12	
Satisfaccion_profesorado	640	4,13	0,042	-365,901	735,8	744,72	
Satisfaccion_compa_2cat	640	4,05	0,044	-366,354	736,71	745,63	
Nivel_estudios_padre_2cat	640	2,92	0,087	-366,552	737,1	746,03	
SEXISM_HOSTIL	640	2,7	0,1	-366,608	737,22	746,14	
Satisfaccion_compa	640	2,37	0,124	-366,755	737,51	746,43	
SEXISM_TOTAL	640	1,49	0,222	-367,052	738,1	747,03	
Situacion_laboral_padre_2cat	640	0,69	0,408	-367,334	738,67	747,59	
MVQ_ACEPTACION	640	0,67	0,413	-367,385	738,77	747,69	
Nivel_estudios_madre_2cat	640	0,3	0,585	-367,508	739,02	747,94	
edad_agrupada	640	3,25	0,196	-366,514	739,03	752,41	
Situacion_laboral_madre_2cat	640	0,27	0,607	-367,526	739,05	747,98	
SEXISM_BENEVOLENTE	640	0,23	0,631	-367,537	739,07	748	
EMP_TOTAL	640	0,17	0,682	-367,557	739,11	748,04	
sex	640	0	0,981	-367,625	739,25	748,17	
Orientacion_sexual	640	16,01	0,014	-362,826	739,65	770,88	
Gender	640	3,5	0,174	-366,909	739,82	753,2	

```
. glm violence_dating i.Child_fis_sex2cat, family(poisson) link(log) vce(robust) eform

Iteration 0:   log pseudolikelihood = -369.01225
Iteration 1:   log pseudolikelihood = -359.85106
Iteration 2:   log pseudolikelihood = -359.83652
Iteration 3:   log pseudolikelihood = -359.83652

Generalized linear models                         No. of obs      =        640
Optimization     : ML                             Residual df     =        638
                                                  Scale parameter =          1
Deviance         =  419.6730436                   (1/df) Deviance =   .6577947
Pearson          =  489.9999978                   (1/df) Pearson  =   .7680251

Variance function: V(u) = u                       [Poisson]
Link function    : g(u) = ln(u)                   [Log]

                                                  AIC             =   1.130739
Log pseudolikelihood = -359.8365218               BIC             =  -3702.744

------------------------------------------------------------------------------
                 |               Robust
 violence_dating |        IRR   Std. Err.      z    P>|z|     [95% Conf. Interval]
-----------------+------------------------------------------------------------
Child_fis_sex2cat|
              S□ |   1.954754   .2727987     4.80   0.000     1.486967    2.569702
           _cons |   .1862955   .0180308   -17.36   0.000     .1541054    .2252096
------------------------------------------------------------------------------
```

Se incluye en el paso siguiente la variable en amarillo

ordenamos de menor a mayor AIC

Variable	N	Chi2	p-value	Log likelihood	AIC	BIC
Child_fis_sex2cat	640	23,07	0	-359,837	723,67	732,6
MVQ_MACHISMO	640	10,32	0,001	-356,547	719,09	732,48
Bullying_victima	640	10,39	0,001	-356,587	719,17	732,56
witness_abuse2	640	12,2	0	-356,863	719,73	733,11
SP_TOTAL	640	8,11	0,004	-356,895	719,79	733,18
Satisfaccion_profesorado_2cat	640	6,37	0,012	-357,59	721,18	734,56
AUT_TOTAL	640	5,98	0,014	-357,736	721,47	734,86
tipo_nac	640	10,86	0,004	-356,755	721,51	739,36
Ciberbullying_victima	640	6,43	0,011	-357,762	721,52	734,91
MVQ_TOTAL	640	5,91	0,015	-357,842	721,68	735,07
Bullying_agresor	640	7,38	0,007	-357,884	721,77	735,15
Satisfaccion_instituto_2cat	640	5,36	0,021	-358,064	722,13	735,51
Relacion_familiar	640	4,51	0,034	-358,382	722,76	736,15
Ciberbullying_agresor	640	5,95	0,015	-358,523	723,05	736,43
Relacion_familiar_2cat	640	3,39	0,066	-358,707	723,41	736,8
Nivel_estudios_padre_2cat	640	3,21	0,073	-358,715	723,43	736,81
Satisfaccion_profesorado	640	2,82	0,093	-358,739	723,48	736,86
Satisfaccion_instituto	640	2,86	0,091	-358,821	723,64	737,03
SEXISM_HOSTIL	640	2,81	0,094	-358,85	723,7	737,08
Satisfaccion_compa_2cat	640	2,71	0,1	-358,979	723,96	737,34
Satisfaccion_compa	640	1,83	0,176	-359,214	724,43	737,81
SEXISM_TOTAL	640	1,62	0,204	-359,252	724,5	737,89
edad_agrupada	640	4,01	0,134	-358,512	725,02	742,87
Situacion_laboral_padre_2cat	640	0,45	0,502	-359,652	725,3	738,69
EMP_TOTAL	640	0,47	0,491	-359,651	725,3	738,69
Nivel_estudios_madre_2cat	640	0,39	0,534	-359,69	725,38	738,76
SEXISM_BENEVOLENTE	640	0,3	0,584	-359,728	725,46	738,84
sex	640	0,02	0,902	-359,831	725,66	739,05
MVQ_ACEPTACION	640	0,02	0,901	-359,831	725,66	739,05
Situacion_laboral_madre_2cat	640	0	0,969	-359,836	725,67	739,06
Gender	640	2,14	0,342	-359,503	727,01	744,85
Orientacion_sexual	640	9,62	0,142	-357,001	730	765,69

Se incluye en el paso siguiente la variable en amarillo

```
. glm violence_dating i.Child_fis_sex2cat c.MVQ_MACHISMO, family(poisson) link(log) vce(robust) eform

Iteration 0:   log pseudolikelihood = -365.95656
Iteration 1:   log pseudolikelihood = -356.56654
Iteration 2:   log pseudolikelihood = -356.54747
Iteration 3:   log pseudolikelihood = -356.54747

Generalized linear models                         No. of obs      =        640
Optimization     : ML                             Residual df     =        637
                                                  Scale parameter =          1
Deviance         =  413.0949401                   (1/df) Deviance =   .6485007
Pearson          =  484.4590154                   (1/df) Pearson  =   .7605322

Variance function: V(u) = u                       [Poisson]
Link function    : g(u) = ln(u)                   [Log]

                                                  AIC             =   1.123586
Log pseudolikelihood =  -356.54747                BIC             =   -3702.86

------------------------------------------------------------------------------
                |               Robust
violence_dating |        IRR   Std. Err.      z    P>|z|     [95% Conf. Interval]
----------------+-------------------------------------------------------------
Child_fis_sex2cat |
             S□ |   1.792715   .2612281     4.01   0.000      1.34734    2.385313
   MVQ_MACHISMO |   1.027884   .0088003     3.21   0.001      1.01078    1.045278
          _cons |   .1456287    .018052   -15.54   0.000     .1142175    .1856783
------------------------------------------------------------------------------
```

ordenamos de menor a mayor AIC

Variable	N	Chi2	p-value	Log likelihood	AIC	BIC
Child_fis_sex2cat	640	23,07	0	-359,837	723,67	732,6
MVQ_MACHISMO	640	10,32	0,001	-356,547	719,09	732,48
Bullying_victima	640	13,86	0	-352,553	713,11	730,95
witness_abuse2	640	13,11	0	-353,508	715,02	732,86
SP_TOTAL	640	5,37	0,02	-354,555	717,11	734,96
Ciberbullying_victima	640	6,04	0,014	-354,621	717,24	735,09
AUT_TOTAL	640	5,1	0,024	-354,713	717,43	735,27
tipo_nac	640	10,17	0,006	-353,725	717,45	739,76
MVQ_ACEPTACION	640	4,23	0,04	-355,064	718,13	735,97
Relacion_familiar	640	3,92	0,048	-355,311	718,62	736,47
Bullying_agresor	640	4,33	0,037	-355,372	718,74	736,59
Satisfaccion_profesorado_2cat	640	3,15	0,076	-355,408	718,82	736,66
Relacion_familiar_2cat	640	3,27	0,071	-355,448	718,9	736,74
Satisfaccion_instituto_2cat	640	3,12	0,078	-355,498	719	736,84
Satisfaccion_compa_2cat	640	1,94	0,163	-355,894	719,79	737,63
Nivel_estudios_padre_2cat	640	1,71	0,19	-355,943	719,89	737,73
Satisfaccion_instituto	640	1,49	0,223	-356,026	720,05	737,9
Nivel_estudios_madre_2cat	640	1,23	0,268	-356,061	720,12	737,97
Ciberbullying_agresor	640	1,82	0,177	-356,098	720,2	738,04
Satisfaccion_compa	640	1,03	0,311	-356,186	720,37	738,22
Satisfaccion_profesorado	640	0,94	0,333	-356,193	720,39	738,23
Situacion_laboral_padre_2cat	640	0,81	0,369	-356,215	720,43	738,28
SEXISM_HOSTIL	640	0,35	0,554	-356,422	720,84	738,69
SEXISM_BENEVOLENTE	640	0,35	0,552	-356,42	720,84	738,69
edad_agrupada	640	3,15	0,207	-355,476	720,95	743,26
Situacion_laboral_madre_2cat	640	0,11	0,736	-356,505	721,01	738,86
EMP_TOTAL	640	0,05	0,831	-356,529	721,06	738,9
sex	640	0	0,954	-356,546	721,09	738,94
SEXISM_TOTAL	640	0	0,989	-356,547	721,09	738,94
Gender	640	2,79	0,248	-355,963	721,93	744,23
Orientacion_sexual	640	13,86	0,031	-352,63	723,26	763,41

Se incluye en el paso siguiente la variable en amarillo

```
Generalized linear models                          No. of obs      =        640
Optimization     : ML                              Residual df     =        636
                                                   Scale parameter =          1
Deviance         =  405.1052604                    (1/df) Deviance =    .636958
Pearson          =  483.9766027                    (1/df) Pearson  =   .7609695

Variance function: V(u) = u                        [Poisson]
Link function    : g(u) = ln(u)                    [Log]

                                                   AIC             =   1.114227
Log pseudolikelihood = -352.5526302                BIC             =  -3704.388

------------------------------------------------------------------------------
                 |               Robust
 violence_dating |        IRR   Std. Err.      z    P>|z|     [95% Conf. Interval]
-----------------+------------------------------------------------------------
Child_fis_sex2cat|
              S□ |   1.657143    .244738     3.42   0.001     1.240648    2.213457
    MVQ_MACHISMO |   1.031084   .0085095     3.71   0.000      1.01454    1.047898
                 |
 Bullying_victima|
           1 vez |   1.762277   .2681748     3.72   0.000     1.307803    2.374685
           _cons |    .129381   .0167932   -15.76   0.000       .10032    .1668605
------------------------------------------------------------------------------
```

ordenamos de menor a mayor AIC

Variable	N	Chi2	p-value	Log likelihood	AIC	BIC
Child_fis_sex2cat	640	23,07	0	-359,837	723,67	732,6
MVQ_MACHISMO	640	10,32	0,001	-356,547	719,09	732,48
Bullying_victima	640	13,86	0	-352,553	713,11	730,95
witness_abuse2	640	11,04	0,001	-349,986	709,97	732,28
SP_TOTAL	640	3,82	0,051	-351,125	712,25	734,56
tipo_nac	640	7,51	0,023	-350,414	712,83	739,6
Satisfaccion_profesorado_2cat	640	2,96	0,085	-351,528	713,06	735,36
Satisfaccion_instituto_2cat	640	2,6	0,107	-351,726	713,45	735,76
AUT_TOTAL	640	2,3	0,129	-351,731	713,46	735,77
MVQ_ACEPTACION	640	2,4	0,122	-351,745	713,49	735,8
Nivel_estudios_padre_2cat	640	2,05	0,152	-351,854	713,71	736,01
Relacion_familiar	640	2,31	0,129	-351,854	713,71	736,02
Ciberbullying_victima	640	2,08	0,149	-351,86	713,72	736,03
Bullying_agresor	640	2,08	0,149	-352,024	714,05	736,35
Relacion_familiar_2cat	640	1,53	0,216	-352,057	714,11	736,42
Nivel_estudios_madre_2cat	640	1,26	0,262	-352,084	714,17	736,48
Ciberbullying_agresor	640	1,66	0,198	-352,158	714,32	736,62
Situacion_laboral_padre_2cat	640	0,93	0,336	-352,165	714,33	736,64
Satisfaccion_instituto	640	1,17	0,278	-352,168	714,34	736,64
Satisfaccion_profesorado	640	0,91	0,341	-352,251	714,5	736,81
SEXISM_BENEVOLENTE	640	0,84	0,359	-352,272	714,54	736,85
Satisfaccion_compa_2cat	640	0,67	0,413	-352,334	714,67	736,98
SEXISM_HOSTIL	640	0,53	0,465	-352,361	714,72	737,03
Satisfaccion_compa	640	0,22	0,638	-352,479	714,96	737,27
Situacion_laboral_madre_2cat	640	0,16	0,687	-352,493	714,99	737,29
EMP_TOTAL	640	0,12	0,732	-352,507	715,01	737,32
sex	640	0	0,981	-352,552	715,1	737,41
SEXISM_TOTAL	640	0,01	0,908	-352,548	715,1	737,4
edad_agrupada	640	2,03	0,362	-351,842	715,68	742,45
Gender	640	2,39	0,303	-352,103	716,21	742,98
Orientacion_sexual	640	9,54	0,145	-349,805	719,61	764,22

Se incluye en el paso siguiente la variable en amarillo

```
Generalized linear models                          No. of obs      =        640
Optimization     : ML                              Residual df     =        635
                                                   Scale parameter =          1
Deviance         =  399.9729157                    (1/df) Deviance =   .6298786
Pearson          =  486.1806376                    (1/df) Pearson  =   .7656388

Variance function: V(u) = u                        [Poisson]
Link function    : g(u) = ln(u)                    [Log]

                                                   AIC             =   1.109333
Log pseudolikelihood = -349.9864579                BIC             =  -3703.059
```

violence_dating	IRR	Robust Std. Err.	z	P>|z|	[95% Conf.	Interval]
Child_fis_sex2cat						
S□	1.536783	.2231776	2.96	0.003	1.156106	2.042807
MVQ_MACHISMO	1.031104	.0083021	3.80	0.000	1.01496	1.047505
Bullying_victima						
1 vez	1.700374	.2563795	3.52	0.000	1.265323	2.285005
witness_abuse2						
S□	1.702068	.2725042	3.32	0.001	1.243649	2.329463
_cons	.124985	.016408	-15.84	0.000	.0966301	.1616602

ordenamos de menor a mayor AIC

Variable	N	Chi2	p-value	Log likelihood	AIC	BIC
Child_fis_sex2cat	640	23,07	0	-359,837	723,67	732,6
MVQ_MACHISMO	640	10,32	0,001	-356,547	719,09	732,48
Bullying_victima	640	13,86	0	-352,553	713,11	730,95
witness_abuse2	640	11,04	0,001	-349,986	709,97	732,28
SP_TOTAL	640	3,31	0,069	-348,734	709,47	736,24
Satisfaccion_profesorado_2cat	640	2,66	0,103	-349,07	710,14	736,91
Satisfaccion_instituto_2cat	640	2,77	0,096	-349,111	710,22	736,99
MVQ_ACEPTACION	640	2,1	0,147	-349,291	710,58	737,35
AUT_TOTAL	640	1,91	0,167	-349,314	710,63	737,4
Situacion_laboral_padre_2cat	640	1,55	0,213	-349,354	710,71	737,48
tipo_nac	640	5,29	0,071	-348,391	710,78	742,01
Nivel_estudios_madre_2cat	640	1,51	0,219	-349,428	710,86	737,62
Ciberbullying_victima	640	1,62	0,203	-349,46	710,92	737,69
Satisfaccion_instituto	640	1,56	0,212	-349,479	710,96	737,73
Relacion_familiar	640	1,64	0,201	-349,49	710,98	737,75
Nivel_estudios_padre_2cat	640	1,34	0,247	-349,531	711,06	737,83
Relacion_familiar_2cat	640	1,03	0,31	-349,654	711,31	738,08
SEXISM_BENEVOLENTE	640	0,99	0,32	-349,662	711,32	738,09
Bullying_agresor	640	1,22	0,27	-349,663	711,33	738,1
Ciberbullying_agresor	640	1,43	0,232	-349,684	711,37	738,14
Satisfaccion_profesorado	640	0,87	0,35	-349,69	711,38	738,15
SEXISM_HOSTIL	640	0,47	0,494	-349,821	711,64	738,41
Satisfaccion_compa_2cat	640	0,43	0,513	-349,85	711,7	738,47
Situacion_laboral_madre_2cat	640	0,36	0,55	-349,857	711,71	738,48
EMP_TOTAL	640	0,16	0,69	-349,925	711,85	738,62
Satisfaccion_compa	640	0,05	0,823	-349,97	711,94	738,71
SEXISM_TOTAL	640	0,03	0,853	-349,975	711,95	738,72
sex	640	0,02	0,879	-349,978	711,96	738,73
edad_agrupada	640	1,19	0,551	-349,574	713,15	744,38
Gender	640	2,61	0,271	-349,607	713,21	744,44
Orientacion_sexual	640	9,63	0,141	-347,307	716,61	765,69

Se incluye en el paso siguiente la variable en amarillo

```
Generalized linear models                          No. of obs      =        640
Optimization     : ML                              Residual df     =        634
                                                   Scale parameter =          1
Deviance         =  397.4686864                    (1/df) Deviance =   .6269222
Pearson          =  487.0858177                    (1/df) Pearson  =   .7682742

Variance function: V(u) = u                        [Poisson]
Link function    : g(u) = ln(u)                    [Log]

                                                   AIC             =   1.108545
Log pseudolikelihood = -348.7343432                BIC             =  -3699.102

------------------------------------------------------------------------------------
                   |               Robust
   violence_dating |       IRR   Std. Err.      z    P>|z|     [95% Conf. Interval]
-------------------+----------------------------------------------------------------
 Child_fis_sex2cat |
                S  |  1.522871   .2186229     2.93   0.003     1.149384    2.017723
      MVQ_MACHISMO |  1.027741   .0084571     3.33   0.001     1.011298    1.044451
                   |
  Bullying_victima |
             1 vez |   1.64611   .2503767     3.28   0.001      1.22177    2.217831
                   |
    witness_abuse2 |
                S  |  1.668869    .264561     3.23   0.001     1.223161    2.276988
          SP_TOTAL |  .9890281   .0059984    -1.82   0.069      .977341    1.000855
             _cons |  .2466159   .0953777    -3.62   0.000     .1155639    .5262835
------------------------------------------------------------------------------------
```

ordenamos de menor a mayor AIC

Variable	N	Chi2	p-value	Log likelihood	AIC	BIC
Child_fis_sex2cat	640	23,07	0	-359,837	723,67	732,6
MVQ_MACHISMO	640	10,32	0,001	-356,547	719,09	732,48
Bullying_victima	640	13,86	0	-352,553	713,11	730,95
witness_abuse2	640	11,04	0,001	-349,986	709,97	732,28
SP_TOTAL	640	3,31	0,069	-348,734	709,47	736,24
Satisfaccion_profesorado_2cat	640	2,27	0,132	-347,95	709,9	741,13
tipo_nac	640	5,75	0,056	-347,038	710,08	745,77
Situacion_laboral_padre_2cat	640	1,63	0,202	-348,048	710,1	741,33
Satisfaccion_instituto_2cat	640	2,13	0,144	-348,064	710,13	741,36
MVQ_ACEPTACION	640	2,04	0,153	-348,069	710,14	741,37
Nivel_estudios_madre_2cat	640	1,41	0,235	-348,215	710,43	741,66
Nivel_estudios_padre_2cat	640	1,29	0,257	-348,3	710,6	741,83
SEXISM_BENEVOLENTE	640	1,04	0,308	-348,391	710,78	742,01
Satisfaccion_instituto	640	0,94	0,332	-348,426	710,85	742,08
Ciberbullying_victima	640	0,84	0,358	-348,452	710,9	742,13
Ciberbullying_agresor	640	1,37	0,242	-348,452	710,9	742,13
Satisfaccion_profesorado	640	0,75	0,388	-348,473	710,95	742,18
Bullying_agresor	640	0,96	0,328	-348,479	710,96	742,19
Relacion_familiar	640	0,8	0,371	-348,485	710,97	742,2
Situacion_laboral_madre_2cat	640	0,39	0,532	-348,595	711,19	742,42
Relacion_familiar_2cat	640	0,39	0,532	-348,608	711,22	742,45
AUT_TOTAL	640	0,31	0,576	-348,629	711,26	742,49
SEXISM_HOSTIL	640	0,24	0,621	-348,649	711,3	742,53
Satisfaccion_compa_2cat	640	0,22	0,639	-348,664	711,33	742,56
SEXISM_TOTAL	640	0,1	0,748	-348,699	711,4	742,63
EMP_TOTAL	640	0,08	0,775	-348,702	711,4	742,63
sex	640	0,02	0,886	-348,727	711,45	742,68
Satisfaccion_compa	640	0	0,966	-348,734	711,47	742,7
edad_agrupada	640	1,64	0,44	-348,176	712,35	748,04
Gender	640	1,9	0,386	-348,412	712,82	748,52
Orientacion_sexual	640	7,83	0,251	-346,463	716,93	770,46

Se incluye en el paso siguiente la variable en amarillo

```
Generalized linear models                          No. of obs      =        640
Optimization     : ML                              Residual df     =        633
                                                   Scale parameter =          1
Deviance         =  395.9007782                    (1/df) Deviance =   .6254357
Pearson          =   484.342392                    (1/df) Pearson  =   .7651539

Variance function: V(u) = u                        [Poisson]
Link function    : g(u) = ln(u)                    [Log]

                                                   AIC             =    1.10922
Log pseudolikelihood = -347.9503891                BIC             =  -3694.209
```

violence_dating	IRR	Robust Std. Err.	z	P>|z|	[95% Conf.	Interval]
Child_fis_sex2cat						
S□	1.518415	.2181417	2.91	0.004	1.145786	2.01223
MVQ_MACHISMO	1.024544	.0087465	2.84	0.005	1.007544	1.041831
Bullying_victima						
1 vez	1.643065	.2514793	3.24	0.001	1.217234	2.217867
witness_abuse2						
S□	1.654218	.2685362	3.10	0.002	1.20341	2.273903
SP_TOTAL	.989608	.0060012	-1.72	0.085	.9779155	1.00144
Satisfaccion_profesorado_2cat						
Buena relaci□n	.8047294	.1159556	-1.51	0.132	.6067335	1.067337
_cons	.2833734	.1114121	-3.21	0.001	.1311287	.6123793

ordenamos de menor a mayor AIC

Variable	N	Chi2	p-value	Log likelihood	AIC	BIC
Child_fis_sex2cat	640	23,07	0	-359,837	723,67	732,6
MVQ_MACHISMO	640	10,32	0,001	-356,547	719,09	732,48
Bullying_victima	640	13,86	0	-352,553	713,11	730,95
witness_abuse2	640	11,04	0,001	-349,986	709,97	732,28
SP_TOTAL	640	3,31	0,069	-348,734	709,47	736,24
Satisfaccion_profesorado_2cat	640	2,27	0,132	-347,95	709,9	741,13
MVQ_ACEPTACION	640	2,62	0,106	-347,093	710,19	745,88
tipo_nac	640	5,91	0,052	-346,179	710,36	750,51
Situacion_laboral_padre_2cat	640	1,69	0,194	-347,206	710,41	746,1
Nivel_estudios_madre_2cat	640	1,41	0,236	-347,432	710,86	746,56
Nivel_estudios_padre_2cat	640	1,46	0,227	-347,459	710,92	746,61
Satisfaccion_instituto_2cat	640	1,13	0,287	-347,588	711,18	746,87
Ciberbullying_victima	640	1,05	0,305	-347,597	711,19	746,89
Ciberbullying_agresor	640	1,25	0,265	-347,681	711,36	747,05
SEXISM_BENEVOLENTE	640	0,73	0,393	-347,705	711,41	747,1
Bullying_agresor	640	0,81	0,367	-347,726	711,45	747,14
Relacion_familiar	640	0,47	0,491	-347,804	711,61	747,3
SEXISM_HOSTIL	640	0,29	0,588	-347,849	711,7	747,39
Situacion_laboral_madre_2cat	640	0,26	0,612	-347,858	711,72	747,41
Satisfaccion_instituto	640	0,24	0,621	-347,875	711,75	747,44
Relacion_familiar_2cat	640	0,18	0,673	-347,893	711,79	747,48
AUT_TOTAL	640	0,15	0,696	-347,9	711,8	747,49
Satisfaccion_compa_2cat	640	0,13	0,714	-347,908	711,82	747,51
Satisfaccion_compa	640	0,1	0,756	-347,919	711,84	747,53
EMP_TOTAL	640	0,06	0,803	-347,926	711,85	747,54
SEXISM_TOTAL	640	0,04	0,846	-347,937	711,87	747,57
sex	640	0,03	0,864	-347,94	711,88	747,57
edad_agrupada	640	1,96	0,376	-347,297	712,59	752,75
Gender	640	2,64	0,267	-347,5	713	753,15
Orientacion_sexual	640	8,11	0,23	-345,625	717,25	775,25

Se incluye en el paso siguiente la variable en amarillo

```
Generalized linear models                         No. of obs      =        640
Optimization     : ML                             Residual df     =        632
                                                  Scale parameter =          1
Deviance         =  394.1858752                   (1/df) Deviance =   .6237118
Pearson          =  481.6133046                   (1/df) Pearson  =   .7620464

Variance function: V(u) = u                       [Poisson]
Link function    : g(u) = ln(u)                   [Log]

                                                  AIC             =   1.109665
Log pseudolikelihood = -347.0929376               BIC             =  -3689.462

------------------------------------------------------------------------------
                              |               Robust
              violence_dating |        IRR   Std. Err.      z    P>|z|     [95% Conf. Interval]
------------------------------+-----------------------------------------------------------------
            Child_fis_sex2cat |
                           S□ |   1.547209   .2226722     3.03   0.002     1.166931    2.051411
                 MVQ_MACHISMO |   1.034703   .0104872     3.37   0.001     1.014352    1.055463
                              |
             Bullying_victima |
                        1 vez |   1.575482   .2443949     2.93   0.003     1.162443    2.135282
                              |
               witness_abuse2 |
                           S□ |   1.629952    .263252     3.02   0.002     1.187677    2.236926
                     SP_TOTAL |   .9898201   .0060705    -1.67   0.095     .9779933     1.00179
                              |
Satisfaccion_profesorado_2cat |
                 Buena relaci□n |   .7836116   .1134311    -1.68   0.092     .5900459    1.040677
               MVQ_ACEPTACION |   .9601415   .0241373    -1.62   0.106     .9139798    1.008635
                        _cons |   .3260656   .1313304    -2.78   0.005      .148069    .7180353
------------------------------------------------------------------------------
```
