# Supplementary material for: Dating violence and associated factors among male and female adolescents in Spain
Source: PLoS One. 2021 Nov 10;16(11):e0258994. doi: 10.1371/journal.pone.0258994 (PMC8580219; doi:10.1371/journal.pone.0258994)
Supplement: S2 File — (PDF) [file pone.0258994.s002.pdf]

ordenamos de menor a mayor AIC

Variable	N	Chi2	p-value	Log likelihood	AIC	BIC
Child_fis_sex2cat	328	10,72	0,001	-184,908	373,82	381,4
Bullying_agresor	328	14,33	0	-185,568	375,14	382,72
Bullying_victima	328	10,13	0,001	-185,646	375,29	382,88
Satisfaccion_instituto_2cat	328	9,53	0,002	-185,749	375,5	383,08
Relacion_familiar_2cat	328	8,84	0,003	-185,811	375,62	383,21
witness_abuse2	328	11,67	0,001	-185,865	375,73	383,32
MVQ_MACHISMO	328	7,57	0,006	-186,215	376,43	384,02
Satisfaccion_profesorado_2cat	328	6,04	0,014	-186,474	376,95	384,53
SP_TOTAL	328	5,47	0,019	-186,513	377,03	384,61
AUT_TOTAL	328	5,28	0,022	-186,653	377,31	384,89
Relacion_familiar	328	5,4	0,02	-186,663	377,33	384,91
MVQ_TOTAL	328	5,46	0,019	-186,811	377,62	385,21
Ciberbullying_victima	328	6,17	0,013	-186,84	377,68	385,27
Satisfaccion_instituto	328	4,11	0,043	-187,076	378,15	385,74
Ciberbullying_agresor	328	6,77	0,009	-187,166	378,33	385,92
Orientacion_sexual	328	22,63	0,001	-182,499	379	405,55
Satisfaccion_profesorado	328	1,53	0,216	-187,916	379,83	387,42
edad_agrupada	328	4,19	0,123	-186,981	379,96	391,34
Nivel_estudios_madre_2cat	328	1,25	0,264	-188,073	380,15	387,73
tipo_nac	328	4,91	0,086	-187,085	380,17	391,55
Nivel_estudios_padre_2cat	328	1,03	0,309	-188,207	380,41	388
MVQ_ACEPTACION	328	0,89	0,345	-188,303	380,61	388,19
SEXISM_HOSTIL	328	0,72	0,398	-188,332	380,66	388,25
Satisfaccion_compa	328	0,3	0,582	-188,48	380,96	388,55
SEXISM_TOTAL	328	0,14	0,712	-188,538	381,08	388,66
Situacion_laboral_padre_2cat	328	0,1	0,75	-188,548	381,1	388,68
Satisfaccion_compa_2cat	328	0,05	0,829	-188,572	381,14	388,73
SEXISM_BENEVOLENTE	328	0,04	0,848	-188,575	381,15	388,74
Situacion_laboral_madre_2cat	328	0,02	0,885	-188,581	381,16	388,75
EMP_TOTAL	328	0,02	0,902	-188,582	381,16	388,75
Gender	328	2,53	0,282	-188,026	382,05	393,43

Se incluye en el paso siguiente la variable en amarillo

```
. glm violence_dating i.Child_fis_sex2cat, family(poisson) link(log) vce(robust) eform

Iteration 0:   log pseudolikelihood = -189.61489
Iteration 1:   log pseudolikelihood = -184.91589
Iteration 2:   log pseudolikelihood = -184.90845
Iteration 3:   log pseudolikelihood = -184.90845

Generalized linear models                         No. of obs      =        328
Optimization     : ML                             Residual df     =        326
                                                  Scale parameter =          1
Deviance         =   215.816905                   (1/df) Deviance =    .662015
Pearson          =  250.9999989                   (1/df) Pearson  =   .7699386

Variance function: V(u) = u                       [Poisson]
Link function    : g(u) = ln(u)                   [Log]

                                                  AIC             =   1.139686
Log pseudolikelihood = -184.9084525               BIC             =  -1672.706

------------------------------------------------------------------------------------
                  |               Robust
  violence_dating |       IRR   Std. Err.      z    P>|z|     [95% Conf. Interval]
------------------+-----------------------------------------------------------------
Child_fis_sex2cat |
               S� |  1.895161   .3701079     3.27   0.001     1.292451    2.778934
            _cons |   .187234   .0254862   -12.31   0.000     .1433903    .2444836
------------------------------------------------------------------------------------
```

ordenamos de menor a mayor AIC

Variable	N	Chi2	p-value	Log likelihood	AIC	BIC
Child_fis_sex2cat	640	23,07	0	-359,837	723,67	732,6
Bullying_agresor	328	9,31	0,002	-182,94	371,88	383,26
MVQ_MACHISMO	328	6,01	0,014	-181,04	370,08	385,25
Bullying_victima	328	4,86	0,027	-181,453	370,91	386,08
Satisfaccion_instituto_2cat	328	4,59	0,032	-181,519	371,04	386,21
witness_abuse2	328	6,98	0,008	-181,558	371,12	386,29
Satisfaccion_profesorado_2cat	328	4,06	0,044	-181,57	371,14	386,31
Relacion_familiar_2cat	328	3,85	0,05	-181,746	371,49	386,66
SP_TOTAL	328	2,98	0,084	-181,826	371,65	386,82
Nivel_estudios_madre_2cat	328	2,42	0,12	-182,016	372,03	387,2
Satisfaccion_instituto	328	2,2	0,138	-182,184	372,37	387,54
AUT_TOTAL	328	1,58	0,208	-182,393	372,79	387,96
Ciberbullying_victima	328	1,67	0,196	-182,438	372,88	388,05
edad_agrupada	328	4,08	0,13	-181,454	372,91	391,87
SEXISM_HOSTIL	328	1,18	0,278	-182,552	373,1	388,28
Satisfaccion_profesorado	328	0,99	0,32	-182,585	373,17	388,34
Relacion_familiar	328	1,06	0,303	-182,613	373,23	388,4
tipo_nac	328	3,89	0,143	-181,685	373,37	392,34
Situacion_laboral_madre_2cat	328	0,45	0,504	-182,767	373,53	388,71
MVQ_ACEPTACION	328	0,45	0,504	-182,83	373,66	388,83
Nivel_estudios_padre_2cat	328	0,28	0,596	-182,843	373,69	388,86
Situacion_laboral_padre_2cat	328	0,27	0,6	-182,851	373,7	388,87
SEXISM_TOTAL	328	0,21	0,65	-182,898	373,8	388,97
Ciberbullying_agresor	328	0,13	0,72	-182,908	373,82	388,99
Satisfaccion_compa_2cat	328	0,07	0,794	-182,918	373,84	389,01
SEXISM_BENEVOLENTE	328	0,09	0,764	-182,937	373,87	389,05
EMP_TOTAL	328	0,05	0,825	-182,946	373,89	389,06
Satisfaccion_compa	328	0	0,999	-182,967	373,93	389,11
Orientacion_sexual	328	15,3	0,018	-178,65	375,3	409,44
Gender	328	1,62	0,446	-182,737	375,47	394,44

Se incluye en el paso siguiente la variable en amarillo

```
end of do-file

. glm violence_dating i.Child_fis_sex2cat i.Bullying_agresor, family(poisson) link(log) vce(robust) eform

Iteration 0:   log pseudolikelihood = -187.47601
Iteration 1:   log pseudolikelihood = -182.94665
Iteration 2:   log pseudolikelihood = -182.93987
Iteration 3:   log pseudolikelihood = -182.93987

Generalized linear models                         No. of obs      =        328
Optimization     : ML                             Residual df     =        325
                                                  Scale parameter =          1
Deviance         =  211.8797317                   (1/df) Deviance =   .6519376
Pearson          =  251.3834424                   (1/df) Pearson  =   .7734875

Variance function: V(u) = u                       [Poisson]
Link function    : g(u) = ln(u)                   [Log]

                                                  AIC             =    1.13378
Log pseudolikelihood = -182.9398659               BIC             =   -1670.85

------------------------------------------------------------------------------------
                  |               Robust
  violence_dating |        IRR   Std. Err.      z    P>|z|     [95% Conf. Interval]
------------------+-----------------------------------------------------------------
Child_fis_sex2cat |
               S� |   1.736407   .3461984     2.77   0.006     1.174741    2.566617
                  |
 Bullying_agresor |
           1 vez  |   2.048074   .4812942     3.05   0.002     1.292155    3.246211
            _cons |   .1800087   .0248142   -12.44   0.000     .1373901    .2358477
------------------------------------------------------------------------------------
```

ordenamos de menor a mayor AIC						
Variable	N	Chi2	p-value	Log likelihood	AIC	BIC
Child_fis_sex2cat	640	23,07	0	-359,837	723,67	732,6
Bullying_agresor	328	9,31	0,002	-182,94	371,88	383,26
MVQ_MACHISMO	328	6,01	0,014	-181,04	370,08	385,25
Bullying_victima	328	4,86	0,027	-181,453	370,91	386,08
Satisfaccion_instituto_2cat	328	4,59	0,032	-181,519	371,04	386,21
witness_abuse2	328	6,98	0,008	-181,558	371,12	386,29
Satisfaccion_profesorado_2cat	328	4,06	0,044	-181,57	371,14	386,31
Relacion_familiar_2cat	328	3,85	0,05	-181,746	371,49	386,66
SP_TOTAL	328	2,98	0,084	-181,826	371,65	386,82
Nivel_estudios_madre_2cat	328	2,42	0,12	-182,016	372,03	387,2
Satisfaccion_instituto	328	2,2	0,138	-182,184	372,37	387,54
AUT_TOTAL	328	1,58	0,208	-182,393	372,79	387,96
Ciberbullying_victima	328	1,67	0,196	-182,438	372,88	388,05
edad_agrupada	328	4,08	0,13	-181,454	372,91	391,87
SEXISM_HOSTIL	328	1,18	0,278	-182,552	373,1	388,28
Satisfaccion_profesorado	328	0,99	0,32	-182,585	373,17	388,34
Relacion_familiar	328	1,06	0,303	-182,613	373,23	388,4
tipo_nac	328	3,89	0,143	-181,685	373,37	392,34
Situacion_laboral_madre_2cat	328	0,45	0,504	-182,767	373,53	388,71
MVQ_ACEPTACION	328	0,45	0,504	-182,83	373,66	388,83
Nivel_estudios_padre_2cat	328	0,28	0,596	-182,843	373,69	388,86
Situacion_laboral_padre_2cat	328	0,27	0,6	-182,851	373,7	388,87
SEXISM_TOTAL	328	0,21	0,65	-182,898	373,8	388,97
Ciberbullying_agresor	328	0,13	0,72	-182,908	373,82	388,99
Satisfaccion_compa_2cat	328	0,07	0,794	-182,918	373,84	389,01
SEXISM_BENEVOLENTE	328	0,09	0,764	-182,937	373,87	389,05
EMP_TOTAL	328	0,05	0,825	-182,946	373,89	389,06
Satisfaccion_compa	328	0	0,999	-182,967	373,93	389,11
Orientacion_sexual	328	15,3	0,018	-178,65	375,3	409,44
Gender	328	1,62	0,446	-182,737	375,47	394,44

Se incluye en el paso siguiente la variable en amarillo

```
end of do-file

. glm violence_dating i.Child_fis_sex2cat i.Bullying_agresor, family(poisson) link(log) vce(robust) eform

Iteration 0:   log pseudolikelihood = -187.47601
Iteration 1:   log pseudolikelihood = -182.94665
Iteration 2:   log pseudolikelihood = -182.93987
Iteration 3:   log pseudolikelihood = -182.93987

Generalized linear models                         No. of obs      =        328
Optimization     : ML                             Residual df     =        325
                                                  Scale parameter =          1
Deviance         =  211.8797317                   (1/df) Deviance =   .6519376
Pearson          =  251.3834424                   (1/df) Pearson  =   .7734875

Variance function: V(u) = u                       [Poisson]
Link function    : g(u) = ln(u)                   [Log]

                                                  AIC             =    1.13378
Log pseudolikelihood = -182.9398659               BIC             =   -1670.85

------------------------------------------------------------------------------------
                  |               Robust
  violence_dating |       IRR   Std. Err.      z    P>|z|     [95% Conf. Interval]
------------------+-------------------------------------------------------------------
Child_fis_sex2cat |
               S� |  1.736407   .3461984     2.77   0.006     1.174741    2.566617
                  |
 Bullying_agresor |
            1 vez |  2.048074   .4812942     3.05   0.002     1.292155    3.246211
            _cons |  .1800087   .0248142   -12.44   0.000     .1373901    .2358477
------------------------------------------------------------------------------------
```

ordenamos de menor a mayor AIC

Variable	N	Chi2	p-value	Log likelihood	AIC	BIC
Child_fis_sex2cat	640	23,07	0	-359,837	723,67	732,6
Bullying_agresor	328	9,31	0,002	-182,94	371,88	383,26
MVQ_MACHISMO	328	6,01	0,014	-181,04	370,08	385,25
Bullying_victima	328	7,08	0,008	-180,237	370,47	389,44
witness_abuse2	328	7,85	0,005	-180,831	371,66	390,63
Relacion_familiar_2cat	328	4,59	0,032	-180,924	371,85	390,81
Satisfaccion_instituto_2cat	328	3,96	0,047	-181,078	372,16	391,12
Nivel_estudios_madre_2cat	328	2,9	0,088	-181,16	372,32	391,29
SP_TOTAL	328	2,63	0,105	-181,289	372,58	391,54
Satisfaccion_profesorado_2cat	328	2,88	0,09	-181,323	372,65	391,61
Relacion_familiar	328	2,47	0,116	-181,535	373,07	392,03
AUT_TOTAL	328	1,95	0,163	-181,612	373,22	392,19
Ciberbullying_victima	328	1,43	0,231	-181,901	373,8	392,77
Satisfaccion_instituto	328	1,02	0,313	-181,91	373,82	392,79
Situacion_laboral_madre_2cat	328	0,83	0,362	-182,012	374,02	392,99
MVQ_ACEPTACION	328	1,02	0,311	-182,008	374,02	392,98
tipo_nac	328	4,03	0,134	-181,026	374,05	396,81
edad_agrupada	328	3,68	0,159	-181,071	374,14	396,9
SEXISM_BENEVOLENTE	328	0,74	0,389	-182,083	374,17	393,13
Satisfaccion_profesorado	328	0,38	0,539	-182,172	374,34	393,31
Situacion_laboral_padre_2cat	328	0,32	0,574	-182,231	374,46	393,43
Nivel_estudios_padre_2cat	328	0,16	0,693	-182,279	374,56	393,52
SEXISM_TOTAL	328	0,16	0,693	-182,281	374,56	393,53
SEXISM_HOSTIL	328	0,06	0,812	-182,314	374,63	393,59
EMP_TOTAL	328	0,04	0,844	-182,317	374,63	393,6
Satisfaccion_compa_2cat	328	0,03	0,853	-182,323	374,65	393,61
Ciberbullying_agresor	328	0,03	0,855	-182,324	374,65	393,61
Satisfaccion_compa	328	0	0,997	-182,334	374,67	393,63
Orientacion_sexual	328	16,24	0,013	-177,858	375,72	413,65
Gender	328	0,96	0,62	-182,205	376,41	399,17

Se incluye en el paso siguiente la variable en amarillo

ordenamos de menor a mayor AIC

Variable	N	Chi2	p-value	Log likelihood	AIC	BIC
Child_fis_sex2cat	640	23,07	0	-359,837	723,67	732,6
MVQ_MACHISMO	640	10,32	0,001	-356,547	719,09	732,48
Bullying_victima	640	13,86	0	-352,553	713,11	730,95
witness_abuse2	640	13,11	0	-353,508	715,02	732,86
SP_TOTAL	640	5,37	0,02	-354,555	717,11	734,96
Ciberbullying_victima	640	6,04	0,014	-354,621	717,24	735,09
AUT_TOTAL	640	5,1	0,024	-354,713	717,43	735,27
tipo_nac	640	10,17	0,006	-353,725	717,45	739,76
MVQ_ACEPTACION	640	4,23	0,04	-355,064	718,13	735,97
Relacion_familiar	640	3,92	0,048	-355,311	718,62	736,47
Bullying_agresor	640	4,33	0,037	-355,372	718,74	736,59
Satisfaccion_profesorado_2cat	640	3,15	0,076	-355,408	718,82	736,66
Relacion_familiar_2cat	640	3,27	0,071	-355,448	718,9	736,74
Satisfaccion_instituto_2cat	640	3,12	0,078	-355,498	719	736,84
Satisfaccion_compa_2cat	640	1,94	0,163	-355,894	719,79	737,63
Nivel_estudios_padre_2cat	640	1,71	0,19	-355,943	719,89	737,73
Satisfaccion_instituto	640	1,49	0,223	-356,026	720,05	737,9
Nivel_estudios_madre_2cat	640	1,23	0,268	-356,061	720,12	737,97
Ciberbullying_agresor	640	1,82	0,177	-356,098	720,2	738,04
Satisfaccion_compa	640	1,03	0,311	-356,186	720,37	738,22
Satisfaccion_profesorado	640	0,94	0,333	-356,193	720,39	738,23
Situacion_laboral_padre_2cat	640	0,81	0,369	-356,215	720,43	738,28
SEXISM_HOSTIL	640	0,35	0,554	-356,422	720,84	738,69
SEXISM_BENEVOLENTE	640	0,35	0,552	-356,42	720,84	738,69
edad_agrupada	640	3,15	0,207	-355,476	720,95	743,26
Situacion_laboral_madre_2cat	640	0,11	0,736	-356,505	721,01	738,86
EMP_TOTAL	640	0,05	0,831	-356,529	721,06	738,9
sex	640	0	0,954	-356,546	721,09	738,94
SEXISM_TOTAL	640	0	0,989	-356,547	721,09	738,94
Gender	640	2,79	0,248	-355,963	721,93	744,23
Orientacion_sexual	640	13,86	0,031	-352,63	723,26	763,41

```
Generalized linear models                         No. of obs      =        640
Optimization     : ML                             Residual df     =        636
                                                  Scale parameter =          1
Deviance         =  405.1052604                   (1/df) Deviance =    .636958
Pearson          =  483.9766027                   (1/df) Pearson  =   .7609695

Variance function: V(u) = u                       [Poisson]
Link function    : g(u) = ln(u)                   [Log]

                                                  AIC             =   1.114227
Log pseudolikelihood = -352.5526302               BIC             =  -3704.388

------------------------------------------------------------------------------
                 |               Robust
 violence_dating |        IRR   Std. Err.      z    P>|z|     [95% Conf. Interval]
-----------------+------------------------------------------------------------
Child_fis_sex2cat|
              S� |   1.657143    .244738     3.42   0.001     1.240648    2.213457
    MVQ_MACHISMO |   1.031084   .0085095     3.71   0.000      1.01454    1.047898
                 |
 Bullying_victima|
           1 vez |   1.762277   .2681748     3.72   0.000     1.307803    2.374685
           _cons |    .129381   .0167932   -15.76   0.000       .10032    .1668605
------------------------------------------------------------------------------
```

Se incluye en el paso siguiente la variable en amarillo

ordenamos de menor a mayor AIC						
Variable	N	Chi2	p-value	Log likelihood	AIC	BIC
Child_fis_sex2cat	640	23,07	0	-359,837	723,67	732,6
MVQ_MACHISMO	640	10,32	0,001	-356,547	719,09	732,48
Bullying_victima	640	13,86	0	-352,553	713,11	730,95
witness_abuse2	640	11,04	0,001	-349,986	709,97	732,28
SP_TOTAL	640	3,31	0,069	-348,734	709,47	736,24
Satisfaccion_profesorado_2cat	640	2,27	0,132	-347,95	709,9	741,13
tipo_nac	640	5,75	0,056	-347,038	710,08	745,77
Situacion_laboral_padre_2cat	640	1,63	0,202	-348,048	710,1	741,33
Satisfaccion_instituto_2cat	640	2,13	0,144	-348,064	710,13	741,36
MVQ_ACEPTACION	640	2,04	0,153	-348,069	710,14	741,37
Nivel_estudios_madre_2cat	640	1,41	0,235	-348,215	710,43	741,66
Nivel_estudios_padre_2cat	640	1,29	0,257	-348,3	710,6	741,83
SEXISM_BENEVOLENTE	640	1,04	0,308	-348,391	710,78	742,01
Satisfaccion_instituto	640	0,94	0,332	-348,426	710,85	742,08
Ciberbullying_victima	640	0,84	0,358	-348,452	710,9	742,13
Ciberbullying_agresor	640	1,37	0,242	-348,452	710,9	742,13
Satisfaccion_profesorado	640	0,75	0,388	-348,473	710,95	742,18
Bullying_agresor	640	0,96	0,328	-348,479	710,96	742,19
Relacion_familiar	640	0,8	0,371	-348,485	710,97	742,2
Situacion_laboral_madre_2cat	640	0,39	0,532	-348,595	711,19	742,42
Relacion_familiar_2cat	640	0,39	0,532	-348,608	711,22	742,45
AUT_TOTAL	640	0,31	0,576	-348,629	711,26	742,49
SEXISM_HOSTIL	640	0,24	0,621	-348,649	711,3	742,53
Satisfaccion_compa_2cat	640	0,22	0,639	-348,664	711,33	742,56
SEXISM_TOTAL	640	0,1	0,748	-348,699	711,4	742,63
EMP_TOTAL	640	0,08	0,775	-348,702	711,4	742,63
sex	640	0,02	0,886	-348,727	711,45	742,68
Satisfaccion_compa	640	0	0,966	-348,734	711,47	742,7
edad_agrupada	640	1,64	0,44	-348,176	712,35	748,04
Gender	640	1,9	0,386	-348,412	712,82	748,52
Orientacion_sexual	640	7,83	0,251	-346,463	716,93	770,46

Se incluye en el paso siguiente la variable en amarillo

```
Generalized linear models                         No. of obs      =        640
Optimization     : ML                             Residual df     =        633
                                                  Scale parameter =          1
Deviance         =  395.9007782                   (1/df) Deviance =   .6254357
Pearson          =   484.342392                   (1/df) Pearson  =   .7651539

Variance function: V(u) = u                       [Poisson]
Link function    : g(u) = ln(u)                   [Log]

                                                  AIC             =    1.10922
Log pseudolikelihood = -347.9503891               BIC             =  -3694.209

------------------------------------------------------------------------------------------------
                              |               Robust
             violence_dating |        IRR   Std. Err.      z    P>|z|     [95% Conf. Interval]
------------------------------+-----------------------------------------------------------------
            Child_fis_sex2cat |
                          S� |   1.518415   .2181417     2.91   0.004     1.145786     2.01223
                 MVQ_MACHISMO |   1.024544   .0087465     2.84   0.005     1.007544    1.041831
                              |
             Bullying_victima |
                       1 vez  |   1.643065   .2514793     3.24   0.001     1.217234    2.217867
                              |
               witness_abuse2 |
                          S� |   1.654218   .2685362     3.10   0.002      1.20341    2.273903
                     SP_TOTAL |    .989608   .0060012    -1.72   0.085     .9779155     1.00144
                              |
Satisfaccion_profesorado_2cat |
               Buena relaci�n |   .8047294   .1159556    -1.51   0.132     .6067335    1.067337
                        _cons |   .2833734   .1114121    -3.21   0.001     .1311287    .6123793
------------------------------------------------------------------------------------------------
```

ordenamos de menor a mayor AIC

Variable	N	Chi2	p-value	Log likelihood	AIC	BIC
Child_fis_sex2cat	640	23,07	0	-359,837	723,67	732,6
MVQ_MACHISMO	640	10,32	0,001	-356,547	719,09	732,48
Bullying_victima	640	13,86	0	-352,553	713,11	730,95
witness_abuse2	640	11,04	0,001	-349,986	709,97	732,28
SP_TOTAL	640	3,31	0,069	-348,734	709,47	736,24
Nivel_estudios_madre_2cat	328	3,32	0,069	-178,953	369,91	392,66
Satisfaccion_instituto_2cat	328	4,18	0,041	-179,021	370,04	392,8
Satisfaccion_profesorado_2cat	328	2,27	0,132	-179,446	370,89	393,65
SP_TOTAL	328	1,96	0,161	-179,466	370,93	393,69
Relacion_familiar_2cat	328	2,24	0,135	-179,517	371,03	393,79
SEXISM_BENEVOLENTE	328	1,59	0,208	-179,742	371,48	394,24
Satisfaccion_instituto	328	1,21	0,271	-179,791	371,58	394,34
AUT_TOTAL	328	1,1	0,294	-179,831	371,66	394,42
Relacion_familiar	328	0,96	0,328	-179,919	371,84	394,6
Situacion_laboral_madre_2cat	328	0,75	0,386	-179,948	371,9	394,66
MVQ_ACEPTACION	328	0,81	0,368	-179,993	371,99	394,74
Situacion_laboral_padre_2cat	328	0,32	0,57	-180,121	372,24	395
Satisfaccion_profesorado	328	0,31	0,575	-180,119	372,24	395
Satisfaccion_compa_2cat	328	0,29	0,59	-180,148	372,3	395,05
Ciberbullying_victima	328	0,28	0,598	-180,152	372,3	395,06
SEXISM_TOTAL	328	0,26	0,61	-180,155	372,31	395,07
SEXISM_HOSTIL	328	0,2	0,655	-180,169	372,34	395,1
Nivel_estudios_padre_2cat	328	0,19	0,666	-180,174	372,35	395,11
Ciberbullying_agresor	328	0,2	0,657	-180,178	372,36	395,11
Satisfaccion_compa	328	0,05	0,824	-180,222	372,44	395,2
EMP_TOTAL	328	0,03	0,868	-180,225	372,45	395,21
tipo_nac	328	2,56	0,278	-179,352	372,7	399,26
edad_agrupada	328	2,5	0,286	-179,354	372,71	399,26
Gender	328	0,94	0,624	-180,065	374,13	400,68
Orientacion_sexual	328	12,48	0,052	-176,679	375,36	417,08
Orientacion_sexual	640	9,63	0,141	-347,307	716,61	765,69

Se incluye en el paso siguiente la variable en amarillo

violence_dating	IRR	Robust Std. Err.	z	P>|z|	[95% Conf.	Interval]
Child_fis_sex2cat						
S�	1.408252	.2960491	1.63	0.103	.9326889	2.126296
Bullying_agresor						
1 vez	1.548593	.384758	1.76	0.078	.9515917	2.520135
MVQ_MACHISMO	1.023407	.0137673	1.72	0.085	.9967765	1.05075
Bullying_victima						
1 vez	1.79037	.382604	2.73	0.006	1.177715	2.721733
Satisfaccion_instituto_2cat						
Me gusta	.6537848	.1358173	-2.05	0.041	.4351171	.9823438
_cons	.1971181	.051268	-6.24	0.000	.1183967	.328181

ordenamos de menor a mayor AIC

Variable	N	Chi2	p-value	Log likelihood	AIC	BIC
Child_fis_sex2cat	640	23,07	0	-359,837	723,67	732,6
MVQ_MACHISMO	640	10,32	0,001	-356,547	719,09	732,48
Bullying_victima	640	13,86	0	-352,553	713,11	730,95
witness_abuse2	640	11,04	0,001	-349,986	709,97	732,28
SP_TOTAL	640	3,31	0,069	-348,734	709,47	736,24
Satisfaccion_profesorado_2cat	640	2,27	0,132	-347,95	709,9	741,13
MVQ_ACEPTACION	640	2,62	0,106	-347,093	710,19	745,88
tipo_nac	640	5,91	0,052	-346,179	710,36	750,51
Situacion_laboral_padre_2cat	640	1,69	0,194	-347,206	710,41	746,1
Nivel_estudios_madre_2cat	640	1,41	0,236	-347,432	710,86	746,56
Nivel_estudios_padre_2cat	640	1,46	0,227	-347,459	710,92	746,61
Satisfaccion_instituto_2cat	640	1,13	0,287	-347,588	711,18	746,87
Ciberbullying_victima	640	1,05	0,305	-347,597	711,19	746,89
Ciberbullying_agresor	640	1,25	0,265	-347,681	711,36	747,05
SEXISM_BENEVOLENTE	640	0,73	0,393	-347,705	711,41	747,1
Bullying_agresor	640	0,81	0,367	-347,726	711,45	747,14
Relacion_familiar	640	0,47	0,491	-347,804	711,61	747,3
SEXISM_HOSTIL	640	0,29	0,588	-347,849	711,7	747,39
Situacion_laboral_madre_2cat	640	0,26	0,612	-347,858	711,72	747,41
Satisfaccion_instituto	640	0,24	0,621	-347,875	711,75	747,44
Relacion_familiar_2cat	640	0,18	0,673	-347,893	711,79	747,48
AUT_TOTAL	640	0,15	0,696	-347,9	711,8	747,49
Satisfaccion_compa_2cat	640	0,13	0,714	-347,908	711,82	747,51
Satisfaccion_compa	640	0,1	0,756	-347,919	711,84	747,53
EMP_TOTAL	640	0,06	0,803	-347,926	711,85	747,54
SEXISM_TOTAL	640	0,04	0,846	-347,937	711,87	747,57
sex	640	0,03	0,864	-347,94	711,88	747,57
edad_agrupada	640	1,96	0,376	-347,297	712,59	752,75
Gender	640	2,64	0,267	-347,5	713	753,15
Orientacion_sexual	640	8,11	0,23	-345,625	717,25	775,25

Se incluye en el paso siguiente la variable en amarillo

```
Generalized linear models                         No. of obs      =        640
Optimization     : ML                             Residual df     =        632
                                                  Scale parameter =          1
Deviance         =  394.1858752                   (1/df) Deviance =   .6237118
Pearson          =  481.6133046                   (1/df) Pearson  =   .7620464

Variance function: V(u) = u                       [Poisson]
Link function    : g(u) = ln(u)                   [Log]

                                                  AIC             =   1.109665
Log pseudolikelihood = -347.0929376               BIC             =  -3689.462

------------------------------------------------------------------------------------------
                              |               Robust
              violence_dating |        IRR   Std. Err.      z    P>|z|     [95% Conf. Interval]
------------------------------+-----------------------------------------------------------
            Child_fis_sex2cat |
                           S� |   1.547209   .2226722     3.03   0.002     1.166931    2.051411
                 MVQ_MACHISMO |   1.034703   .0104872     3.37   0.001     1.014352    1.055463
                              |
             Bullying_victima |
                       1 vez  |   1.575482   .2443949     2.93   0.003     1.162443    2.135282
                              |
               witness_abuse2 |
                           S� |   1.629952    .263252     3.02   0.002     1.187677    2.236926
                     SP_TOTAL |   .9898201   .0060705    -1.67   0.095     .9779933     1.00179
                              |
Satisfaccion_profesorado_2cat |
               Buena relaci�n |   .7836116   .1134311    -1.68   0.092     .5900459    1.040677
               MVQ_ACEPTACION |   .9601415   .0241373    -1.62   0.106     .9139798    1.008635
                        _cons |   .3260656   .1313304    -2.78   0.005      .148069    .7180353
------------------------------------------------------------------------------------------
```

```
Generalized linear models                         No. of obs      =        328
Optimization     : ML                             Residual df     =        322
                                                  Scale parameter =          1
Deviance         =  203.1814991                   (1/df) Deviance =   .6309984
Pearson          =  248.9408923                   (1/df) Pearson  =   .7731084

Variance function: V(u) = u                       [Poisson]
Link function    : g(u) = ln(u)                   [Log]

                                                  AIC             =   1.125553
Log pseudolikelihood = -178.5907495               BIC             =  -1662.169
```

violence_dating	IRR	Robust Std. Err.	z	P>|z|	[95% Conf.	Interval]
Child_fis_sex2cat						
S�	1.304796	.2763021	1.26	0.209	.8615753	1.976023
MVQ_MACHISMO	1.030883	.0130029	2.41	0.016	1.00571	1.056686
Bullying_victima						
1 vez	1.841105	.3994329	2.81	0.005	1.203394	2.816755
witness_abuse2						
S�	1.698182	.4066779	2.21	0.027	1.062037	2.715367
Satisfaccion_instituto_2cat						
Me gusta	.6561491	.1377855	-2.01	0.045	.4347682	.9902555
_cons	.1838775	.0498916	-6.24	0.000	.1080368	.3129577
